# Serum Acylated Ghrelin Concentrations in Response to Short-Term Overfeeding in Normal Weight, Overweight, and Obese Men

**DOI:** 10.1371/journal.pone.0045748

**Published:** 2012-09-27

**Authors:** Danny Wadden, Farrell Cahill, Peyvand Amini, Edward Randell, Sudesh Vasdev, Yanqing Yi, Weizhen Zhang, Guang Sun

**Affiliations:** 1 Division of Medicine, Faculty of Medicine, Memorial University of Newfoundland, St. John’s, Newfoundland, Canada; 2 Discipline of Laboratory Medicine, Memorial University of Newfoundland, St. John’s, Newfoundland, Canada; 3 Department of Physiology and Pathophysiology, School of Basic Medical Sciences, Peking University, Beijing, China; University of Santiago de Compostela School of Medicine - CIMUS, Spain

## Abstract

**Background:**

Ghrelin, an orexigenic gut hormone secreted primarily from the stomach, is involved in energy homeostasis. However, little data is available regarding its response to energy surplus and the development of human obesity.

**Objective:**

The present study investigated the response of circulating acylated ghrelin to a 7-day positive energy challenge.

**Design:**

A total of 68 healthy young men were overfed 70% more calories than required, for 1-week. Subjects were classified based on percent body fat (measured by dual-energy X-ray absorptiometry) as normal weight, overweight, and obese. Serum acylated ghrelin concentration was measured before and after the positive energy challenge. Additionally, the relationship between acylated ghrelin and obesity-related phenotypes including weight, body mass index, percent body fat, cholesterol, HDL-c, LDL-c, glucose, insulin and homeostasis model assessment of insulin resistance and β-cell function at baseline and change due to overfeeding, were assessed.

**Results:**

Contrary to our expectations, serum acylated ghrelin was significantly increased in response to overfeeding and the increase was independent of obesity status. There was no significant difference in fasting acylated ghrelin between normal weight, overweight, and obese men at baseline. Acylated ghrelin was negatively correlated with weight and BMI for normal weight and with BMI in overweight men. Also ghrelin was correlated with change in weight and BMI in overweight (negative relationship) and obese (positive relationship) groups.

**Conclusion:**

Our results showed that circulating acylated ghrelin was increased after a 7-day positive energy challenge regardless of adiposity status. However, acylated ghrelin was correlated with change in weight and BMI in opposing directions, in overweight and obese subjects respectively, thus dependent on obesity status.

## Introduction

Although it has been well-established that appetite is controlled through complex mechanisms mainly in the central nervous system (CNS), appetite-regulating hormones secreted from the periphery, including the gastrointestinal tract (gut), communicate with the CNS to play an important role in energy homeostasis [1]–[3]. Ghrelin, an endogenous ligand of the growth hormone secretagogue receptor (GHS-R), is the only known orexigenic gut hormone, which increases appetite and food intake [4]–[6]. Ghrelin is found in both the gastrointestinal tract and hypothalamus, though it is primarily synthesized and released from X/A like cells of the gastric mucosa of the stomach [4], [7]–[9]. Both acylated and non-acylated forms of ghrelin exist, however the active form is n-octanoylated on the third amino acid (serine) residue [4], [10], [11]. Ghrelin is thought to be a meal initiator: concentration rises prior to feeding, and continually decreases to a minimum one-hour after the meal [12], [13]. The postprandial suppression of circulating ghrelin is proportional to the caloric content of the consumed meal [14].

Many studies have investigated ghrelin’s role in the regulation of appetite and energy homeostasis. Administration of ghrelin in both rats and humans has been shown to increase appetite and food intake [6], [15]–[18]. However in humans, most studies have found a negative relationship between ghrelin and adiposity: higher circulating ghrelin concentrations in lean individuals as compared to obese [19]–[24]. In contrast, other studies have found positive relationships between acylated ghrelin concentrations and markers of adiposity [25] or a negative relationship with BMI and no relationship to body fat [26]. Additionally, a growing body of evidence shows that ghrelin might have a role in insulin resistance and development of type 2 diabetes. Animal studies have shown that ghrelin can inhibit insulin secretion, but increases glucagon secretion from pancreatic islet cells [27]. Moreover human studies reveal that patients with insulin resistance have lower ghrelin concentrations compared with insulin-sensitive patients [28].

Circulating ghrelin concentration seems to be influenced through dietary regulation and specific nutrient intakes. For example, a high-fat meal has been shown to decrease circulating ghrelin concentration in humans [29], [30]. Moreover, our laboratory and others have revealed changes in nutritional status, such as overfeeding influence adipokine and gut hormone concentrations [31], [32], adipose tissue metabolism [33], [34] and genomic expression [35]. Intervention based studies, observing nutritional regulation via a positive energy challenge, are also important in understanding the role of ghrelin in the development of human obesity where energy surplus is the major driving factor [36]–[40]. Currently, data is missing in this aspect.

Our current research goal was to investigate the potential role of acylated ghrelin on the development of human obesity by examining: 1) the response of fasting serum acylated ghrelin concentrations in normal weight, overweight, and obese males to short-term overfeeding; 2) fasting serum acylated ghrelin concentrations in each adiposity group before and after overfeeding; and 3) the relationships of fasting serum acylated ghrelin concentrations with fasting glucose and insulin concentrations, blood lipids, and insulin resistance state, before and after overfeeding.

## Materials and Methods

### Ethics Statement

All participants provided informed and written consent. The current study received ethical approval from the Human Investigations Committee for the Faculty of Medicine, Memorial University of Newfoundland, St. John’s, Newfoundland and Labrador, Canada.

### Subjects

A total of 68 young males from the Canadian province of Newfoundland and Labrador were recruited to study the effects of an acute positive energy balance on metabolism and various endocrine factors. Participation criteria included: 1) age 19–29 years; 2) at least of 3^rd^-generation Newfoundland descent; 3) no serious cardiovascular, metabolic, or endocrine diseases; 4) no medications intended for lipid metabolism change; and 5) a stable 6-month reported body weight value (±2.5 kg). Recruited subjects were asked to refrain from 1) consuming alcoholic or additional calorie-containing beverages and from 2) taking any drugs or medication, throughout the duration of the research study.

### Serum Measurements

Blood samples were collected from all subjects both before and after completion of the positive energy challenge (explained below). Serum was prepared from clotted samples collected after a 12 hour fast which were stored at −80°C until time of further analysis. Serum acylated ghrelin concentrations were measured in duplicate with enzymeimmunosorbent assay (EIA) kits (Bertin Pharma; Montigny le Bretonneux, France). Acylated ghrelin analyses were performed on ice. The concentrations of serum total cholesterol, high-density lipoprotein (HDL) cholesterol, triacylglycerols (TG), and glucose were analyzed using Synchron reagents by an Lx20 clinical chemistry analyzer (Beckman Coulter Inc., CA, USA). Low-density lipoprotein (LDL) cholesterol was calculated by the Friedwald equation: total cholesterol – HDL – TG/2.2 (which is a reliable estimate in the absence of severe hypertriglyceridemia >4.6 mmol/L). Serum insulin was evaluated using an Immulite 2500 immunoassay analyzer (Siemens, Los Angeles, CA). Insulin resistance and pancreatic β-cell function were assessed using the homeostasis model assessment (HOMA). Insulin resistance was quantified using HOMA-IR [insulin (mU/L) × glucose (mmol/L)/22.5)] while β-cell function was measured using HOMA-β [20× insulin (mU/L/(glucose (mmol/L)-3.5)].

### Body Composition Assessment

Dual-energy X-ray absorptiometry (DXA Lunar Prodigy; GE Medical Systems, Madison, WI) was used to measure body composition (lean tissue, body fat, and bone mineral composition). Following a method previously described by us [41] the participant was scanned in a supine position after a 12-hour fast and the following parameters were determined from the DXA scan: total percent body fat (%BF), percent trunk fat (%TF), percent android fat (%AF), and percent gynoid fat (%GF). All measurements were performed before overfeeding and on the day after overfeeding.

### Anthropometric Measurements

Measurements of body weight, height, waist circumference, and hip circumference were performed after a12-hour fasting period. Body weight was measured to the nearest 0.1 kilogram on a platform manual scale balance (Health O Meter, Bridgeview, IL) with subjects wearing a standardized hospital gown. A fixed stadiometer was used to measure height to the nearest 0.1 centimeters (shoes removed). A flexible measuring tape was employed to measure waist circumference to the nearest 0.1 centimeters at the level of the umbilicus. The same procedure was used to measure hip circumference at the level of largest circumference between the waist and thighs. Body mass index (BMI) was calculated by dividing participants’ weight in kilograms by the square of height in centimeters [(weight-kg)/(height-m)^2^].

### Overfeeding Protocol

Participants enrolled into a 7-day positive energy challenge and were required to consume 70% more calories than their normal food intake. Throughout the challenge period, subjects followed a diet consisting of 15% protein, 35% fat, and 50% carbohydrates, thus mimicking typical North American dietary patterns. A 7-day positive energy challenge was selected to ensure that metabolic changes were induced throughout its duration. Individual energy requirements were recorded and estimated before commencing the overfeeding protocol by the use of three 24-hour recalls and a 30-day dietary inventory. For one week at time 0900, 1200, and 1700, participants were offered meals of which caloric and macronutrient content was assessed using FOOD PROCESSOR SQL software (version 9.5.0.0; ESHA Research, Salem, OR). The average baseline energy intake before and during overfeeding were 2969 kcal and 5471 kcal, respectively. A detailed overfeeding protocol has been described in our previously published papers [33], [34].

### Statistical Analysis

Data are presented as means ± SE unless otherwise stated. Prior to analysis, data that showed a skewed distribution was logarithmically transformed to approximate a normal distribution. Concentration data for serum acylated ghrelin, triacylglycerols, insulin, and HOMA-IR and HOMA-β, all at baseline, after, and change during overfeeding were log transformed prior to analysis. Using criteria suggested by Bray [42] subjects were classified as either normal weight, overweight or obese based upon %BF (8–20.9, 21–25.9 and ≥26%, respectively). Subjects were also classified using the World Health Organization BMI-based classification (BMIs of ≤24.9, 25.0–29.9, and ≥30 kg/m^2^ for normal weight, overweight, and obese respectively). Because of its more accurate classification of obesity status, Bray Criteria was used for subsequent analyses.

Using a two-factor analysis of variance (ANOVA) with repeated measures before and after overfeeding, differences in physical and biochemical variables in response to overfeeding were assessed between the three groups. One-factor analysis of variance was used to compare baseline values between the three adiposity groups. Variables which showed a significant overfeeding-adiposity interaction underwent within-group analysis of the response to overfeeding using a paired t-test. In both one factor and two-factor ANOVA analyses, Bonferroni post hoc tests were employed.

Initial Pearson’s correlation analyses were performed to assess relationships between fasting acylated ghrelin concentration and physical/biochemical variables of interest. Next we performed three correlation analyses: 1) baseline acylated ghrelin concentration was compared with all variables at baseline and 2) baseline acylated ghrelin concentration was compared with changes in all variables in response to overfeeding thus examining if baseline acylated ghrelin could predict the changes in related physical and biochemical markers. All statistical analyses were completed using SPSS, version 19.0 (SPSS Inc, Chicago) in which: 1) all tests were two-sided; and 2) a P value <0.05 was deemed statistically significant.

## Results

### Descriptive Statistics

Basal physical and biochemical characteristics of subjects are shown in **Table 1**. Differences in body composition glucose, and lipid metabolism between normal-weight, overweight, and obese (based on %BF) young healthy men were previously described by us [31], [33]–[35].

**Table 1:** Physical and Biochemical Characteristics of Subjects at baseline and in response to 7-days of overfeeding^1^.

	Entire Cohort (n = 68)						Normal Weight (n = 26)						Overweight (n = 14)						Obese (n = 28)					
	Pre			Post			Pre			Post			Pre			Post			Pre			Post		
Age	23.18	±	0.38	NA			23.75	±	0.72	NA			21.97	±	0.83	NA			23.25	±	0.49	NA		
Height (cm)	179.11	±	0.78	NA			179.04	±	1.29	NA			179.62	±	1.28	NA			178.91	±	1.34	NA		
Weight (kg)^4, 5^	82.18	±	1.80	84.43	±	1.85	72.86	±	1.77	75.06	±	1.84	77.81	±	1.14	79.39	±	1.14	93.01	±	2.95	95.65	±	3.03
BMI (kg/m^2^)^4, 5^	25.64	±	0.56	26.35	±	0.58	22.72	±	0.50	23.42	±	0.53	24.13	±	0.36	24.63	±	0.39	29.10	±	0.92	29.93	±	0.95
Percent body fat^2, 6^	23.23	±	1.03	23.49	±	0.97	14.69	±	0.66	15.48	±	0.67	22.54	±	0.22	22.82	±	0.28	31.51	±	0.95	31.26	±	0.89
Percent trunk fat^2, 6^	26.08	±	1.13	26.41	±	1.06	16.59	±	0.73	17.62	±	0.75	25.39	±	0.50	25.79	±	0.59	35.22	±	1.02	34.89	±	0.96
Percent android fat^2, 5^	29.74	±	1.34	30.46	±	1.33	19.09	±	0.89	20.01	±	0.98	28.84	±	0.68	29.46	±	0.73	40.47	±	1.38	41.06	±	1.30
Percent gynoid fat	28.12	±	0.99	28.36	±	0.94	20.42	±	0.96	20.94	±	0.87	27.45	±	0.48	28.23	±	0.47	35.88	±	0.89	35.58	±	0.87
Total cholesterol (mmol/L)^5^	4.50	±	0.10	4.73	±	0.10	4.37	±	0.18	4.67	±	0.17	4.63	±	0.24	4.73	±	0.28	4.56	±	0.14	4.79	±	0.15
HDL cholesterol (mmol/L)^5^	1.30	±	0.04	1.40	±	0.03	1.38	±	0.06	1.47	±	0.05	1.39	±	0.07	1.43	±	0.07	1.19	±	0.05	1.31	±	0.05
LDL cholesterol (mmol/L)	2.72	±	0.08	2.75	±	0.08	2.59	±	0.14	2.66	±	0.13	2.82	±	0.20	2.83	±	0.24	2.79	±	0.13	2.79	±	0.11
Triglycerols (mmol/L)^4, 5^	1.09	±	0.07	1.44	±	0.17	0.89	±	0.06	1.18	±	0.16	0.92	±	0.09	1.01	±	0.14	1.37	±	0.13	1.91	±	0.36
Glucose (mmol/L)	5.11	±	0.06	5.10	±	0.06	4.97	±	0.08	5.02	±	0.10	5.03	±	0.10	5.09	±	0.15	5.28	±	0.12	5.17	±	0.10
Insulin (pmol/L)^3, 5^	70.82	±	8.68	89.24	±	8.13	43.34	±	4.67	64.96	±	4.65	69.51	±	18.49	88.85	±	23.07	97.00	±	17.35	111.98	±	14.57
HOMA-IR^3, 5^	2.47	±	0.35	3.00	±	0.30	1.40	±	0.16	2.12	±	0.17	2.36	±	0.72	2.95	±	0.79	3.51	±	0.72	3.85	±	0.56
HOMA-β^3, 5^	117.65	±	9.20	163.29	±	12.83	84.13	±	7.69	128.08	±	9.56	120.21	±	19.80	175.91	±	43.72	147.49	±	17.13	189.67	±	19.54
Serum ghrelin (ng/L)^5^	286.51	±	52.30	348.65	±	63.02	222.19	±	41.60	226.14	±	29.26	274.91	±	92.44	284.14	±	88.18	352.03	±	112.42	494.66	±	141.23

1 All values are mean ± SE. Homeostasis model assessment of insulin resistance (HOMA-IR) and of B cell function (HOMA-β); NA, not applicable. Subjects were classifed based on adiposity recommendations by Bray as normal weight (8–20.9%), overweight (21–25.9%), or obese (>26%). Obesity status and overfeeding response were analyzed by 2-factor mixed-model ANOVA for repeated measures (IBM SPSS Statistics 19).

2 Significant difference between normal weight, overweight, and obese subjects at baseline, P<0.05 (one-factor ANOVA with Bonferroni correction).

3 Significant difference between normal weight and obese subjects at baseline, P<0.05 (one-factor ANOVA with Bonferroni correction).

4 Significant difference between obese and normal weight and obese and overweight, at baseline, P<0.05 (one-factor ANOVA with Bonferroni correction).

5 Significant difference due to overfeeding, P<0.05 (2-factor mixed-model ANOVA).

6 Significant overfeeding x adiposity status interaction, P<0.05 (2-factor mixed-model ANOVA with Bonferroni correction).

Baseline fasting acylated ghrelin concentrations (mean ± S.E.) for normal weight, overweight, and obese individuals were 222.19±41.60, 274.91±92.4, and 352.03±112.4 ng/L, respectively. There was no statistically significant difference between adiposity groups for acylated ghrelin concentration at baseline. Similar results were obtained when participants were divided into normal weight, overweight and obese groups based on the World Health Organization’s BMI classification (data not shown).

Changes in body composition and phenotypes of glycemic control and lipid metabolism in response to the 7-day overfeeding challenge are also described in Table 1. Within all adiposity groups, the positive energy challenge increased body composition, serum lipids, insulin resistance, and pancreatic beta cell function, which was previously described by us [31], [33]–[35].

In response to the 7-day overfeeding challenge, circulating acylated ghrelin concentration significantly increased by 62.14 ng/L in the entire cohort (P = 0.042). However, no adiposity-overfeeding interaction was present; the response of acylated ghrelin to overfeeding was not significantly different between normal weight, overweight, and obese groups (P = 0.523).

### Correlations of Acylated Ghrelin with Adiposity and Phenotypes of Glucose and Lipid Metabolism

Partial correlation analyses, controlling for age, were used to assess the relationships between baseline fasting acylated ghrelin concentration and baseline phenotypes of adiposity, and serum glucose and lipid concentrations (**Table 2**). For normal weight subjects, acylated ghrelin was negatively correlated with weight and BMI (P<0.05). Furthermore, fasting glucose concentration was positively correlated with acylated ghrelin concentrations. The significant negative correlation between acylated ghrelin and BMI was also observed in overweight subjects, but not in obese subjects. LDL cholesterol was negatively correlated with acylated ghrelin concentration only in obese subjects.

**Table 2:** Partial correlations of baseline variables related to baseline fasting serum ghrelin concentrationa,b.

	All Subjects (n = 68)		Normal Weight (n = 26)		Overweight (n = 14)		Obese (n = 28)	
	*r*	*P*	*r*	*P*	*r*	*P*	*r*	*P*
Weight (kg)	−0.012	NS	−0.431	0.032	−0.234	NS	0.089	NS
BMI (kg/m^2^)	−0.037	NS	−0.404	0.045	−0.667	0.013	0.067	NS
Percent body fat	−0.007	NS	−0.226	NS	−0.440	NS	−0.086	NS
Percent trunk fat	−0.034	NS	−0.277	NS	−0.425	NS	−0.130	NS
Percent android fat	−0.035	NS	−0.169	NS	−0.548	NS	−0.112	NS
Percent gynoid fat	0.070	NS	−0.096	NS	0.137	NS	0.122	NS
Total cholesterol	−0.138	NS	0.392	NS	−0.370	NS	−0.353	NS
HDL cholesterol	−0.148	NS	0.274	NS	−0.438	NS	−0.290	NS
LDL cholesterol	−0.178	NS	0.292	NS	−0.293	NS	−0.436	0.023
Triacylglycerols	0.145	NS	0.213	NS	−0.073	NS	0.172	NS
Glucose	−0.015	NS	0.473	0.017	−0.140	NS	−0.185	NS
Insulin	0.069	NS	0.187	NS	0.192	NS	−0.071	NS
HOMA-IR	0.061	NS	0.231	NS	0.152	NS	−0.092	NS
HOMA-β	0.097	NS	−0.034	NS	0.384	NS	0.036	NS

a Partial correlations controlled for age were used to screen for variables related to fasting ghrelin (P<0.05) (IBM SPSS Statistics 19).

b Homeostasis model assessment of insulin resistance (HOMA-IR) and of B cell function (HOMA-β); NS, non-significant. Subjects were classifed based on adiposity recommendations by Bray as normal weight (8–20.9%), overweight (21–25.9%), or obese (>26%).

Additionally, partial correlation analyses examining the relationship between baseline acylated ghrelin concentration and changes in variables after the overfeeding period were used to examine if acylated ghrelin concentration could predict variable change under a positive energy challenge (**Table 3**). Significant negative relationships were found for the overweight group, between baseline acylated ghrelin concentration and change in body weight and BMI. However, the relationship between baseline acylated ghrelin and change in body weight and BMI, were positive for obese subjects.

**Table 3:** Partial correlations of change in variables related to baseline fasting serum ghrelin concentrationa,b.

	All Subjects (n = 68)		Normal Weight (n = 26)		Overweight (n = 14)		Obese (n = 28)	
	*r*	*P*	*r*	*P*	*r*	*P*	*r*	*P*
Weight (kg)	0.217	NS	0.208	NS	−0.590	0.034	0.618	0.001
BMI (kg/m^2^)	0.218	NS	0.227	NS	−0.599	0.031	0.616	0.001
Percent body fat	−0.017	NS	0.297	NS	−0.170	NS	−0.145	NS
Percent trunk fat	−0.042	NS	0.235	NS	−0.086	NS	−0.159	NS
Percent android fat	0.084	NS	0.246	NS	0.159	NS	−0.080	NS
Percent gynoid fat	−0.047	NS	0.017	NS	−0.298	NS	0.098	NS
Total cholesterol	0.022	NS	−0.056	NS	−0.043	NS	0.124	NS
HDL cholesterol	0.006	NS	−0.167	NS	−0.125	NS	0.185	NS
LDL cholesterol	0.041	NS	−0.134	NS	0.026	NS	0.202	NS
Triacylglycerols	−0.016	NS	0.233	NS	−0.108	NS	−0.103	NS
Glucose	−0.026	NS	−0.124	NS	−0.169	NS	0.106	NS
Insulin	−0.082	NS	0.044	NS	−0.178	NS	−0.135	NS
HOMA-IR	−0.080	NS	0.013	NS	−0.206	NS	−0.086	NS
HOMA-β	−0.061	NS	0.155	NS	−0.012	NS	−0.343	NS

a Partial correlations controlled for age were used to screen for variables related to fasting ghrelin (P<0.05) (IBM SPSS Statistics 19).

b Homeostasis model assessment of insulin resistance (HOMA-IR) and of B cell function (HOMA-β); NS, non-significant. Subjects were classifed based on adiposity recommendations by Bray as normal weight (8–20.9%), overweight (21–25.9%), or obese (>26%).

### Acylated Ghrelin Tertiles: Correlations of Acylated Ghrelin with Phenotypes of Adiposity and Concentrations of Glucose and Lipids

We repeated all of the above analyses after participants were divided into acylated ghrelin tertile subgroups (low, medium, and high ghrelin; data not shown). Only one baseline correlation existed within the analysis. For the high ghrelin subgroup, baseline acylated ghrelin was positively correlated with baseline fasting triacylglycerols. No significant relationships were found when partial correlation analyses were performed examining baseline acylated ghrelin concentrations and changes in the aforementioned variables.

## Discussion

The most important finding from the present study was the discovery of the significant increase in serum acylated ghrelin concentration after the 7-day positive energy challenge. Conventional knowledge would lead us to expect that to counteract a positive energy balance (thus limiting caloric intake) during overfeeding, secretion of ghrelin would be diminished to decrease appetite. However, the results from our study was surprisingly the opposite; acylated ghrelin concentration significantly increased after the 7-day overfeeding challenge regardless of adiposity status. Pathologically high circulating concentrations of ghrelin are known to occur in the inherited disease of Prader-Willi Syndrome, which is characterized by uncontrollable appetite [43]–[45], but also in cases of anorexia nervosa, where there is little appetite [46], [47].

At the present time, results are not clear based on limited studies examining the responsiveness of ghrelin to a positive energy challenge. Moreover, the few available studies with positive energy challenge interventions differ in many aspects including length of overfeeding, macronutrient composition, amount of food consumed above daily caloric requirements, and also the physical and demographic characteristics of the study participants. Hagobian et al. overfed 9 healthy subjects (6 men and 3 women) 25% more calories than required for weight maintenance for 3 days, retaining a macronutrient composition of 56% carbohydrate, 29% fat, and 15% protein, and found no significant change in circulating ghrelin concentration [38]. Votruba et al. overfed 69 non-diabetic, mainly obese individuals (40 men and 29 women) 60% more calories than required at baseline from a “vending machine diet” over a 3-day period [37]. Circulating ghrelin concentration did not change through the 3-day duration. A 5-day high fat (60% fat, 32.5% carbohydrates and 7.5% protein) overfeeding study (50% more calories than required) found a non-significant increase in circulating ghrelin in a cohort of 26 healthy young men [36]. Robertson et al. overfed 6 healthy lean male subjects by ∼692.7 kcal/day with a high fat diet ranging from 29–45% calories from fat. This study detected no significant change in fasting ghrelin, however, postprandial ghrelin was suppressed to a greater extent following the oral fat tolerance test [39]. Finally, a long-term overfeeding study of 100-day in which twelve pairs of identical twins consumed an excess of 84,000 kilocalories (50% carbohydrates, 35% fat, and 15% protein) revealed a non-significant decrease in plasma ghrelin concentration [40]. Our current study using a typical North American diet (15% protein, 35% fat, and 50% carbohydrates) and a homogenous large sample size consisting of only young male university students, detected a significant increase in circulating acylated ghrelin after 7 days of overfeeding. The reason for the increased acylated ghrelin concentration after the positive energy challenge is not clear. In animal studies, the infusion of ghrelin has been shown to result in inhibition of beta-cell function and insulin secretion, but also an increase in insulin sensitivity [27], [48], [49]. Additionally in cross-sectional human studies, fasting ghrelin is negatively associated with insulin resistance [50]. It is therefore possible that the increased ghrelin secretion due to overfeeding was a counteractive response to the rising insulin resistance after the 7-day overfeeding, though this is purely speculative as multiple factors must be in play. However, the role of this increased response by ghrelin in long-term energy homeostasis, including the development of human obesity, warrants further study.

Although it has been reported that circulating ghrelin is higher in lean individuals than overweight and obese individuals [19]–[24], our cohort showed no significant difference in circulating acylated ghrelin among the three adiposity groups. However, when subjects were divided according to adiposity status, BMI was negatively correlated with acylated ghrelin concentration in both normal weight and overweight subjects. This is in line with the observation that ghrelin may help maintain healthy body weight and BMI. The reason why such correlation was not observed in obese subjects is unclear. It is possible that the normal hormonal mechanism of ghrelin was dysfunctional in the obese state, as seen for many other hormones [51], [52]. This was further demonstrated by the differences in correlations found in overweight and obese subjects, between baseline acylated ghrelin and change in weight and BMI. Overweight subjects showed a negative relationship, while obese subjects showed a positive relationship, between fasting baseline acylated ghrelin and change in body weight and BMI. Thus higher baseline acylated ghrelin predicts low weight gain and BMI in the overweight group, but the opposite is true of the obese group. This may predispose to further weight gain in obese people.

Additionally, we sought to examine the association of acylated ghrelin with serum indices of lipid and glucose metabolism. The associations between ghrelin and circulating lipids/glucose seem to be affected by obesity status. We observed that only baseline acylated ghrelin concentration was positively associated with fasting glucose concentration in normal weight subjects. Additionally in obese subjects, acylated ghrelin was negatively related to circulating LDL cholesterol concentration. Why these relationships were found in specific adiposity groups is unknown. Evidently, more studies are warranted to further grasp the physiological association of ghrelin with glucose and lipid metabolism.

The present study is not without limitations. We only studied young men of the same ethnicity thus limiting its potential application to other ethnic, age, or female groups. Future large scale studies including females and a wider age range are needed to further understand the response of ghrelin to a positive energy challenge. Additionally, it must be noted that ghrelin concentrations are affected by meals [53]. However in our investigation, the relationship between ghrelin and aforementioned physiological measurements were completed in a fasting state. Data of area under the curve after a standardized meal may provide additional useful information.

In summary, the response of acylated ghrelin to a short-term positive energy challenge was studied in 68 young healthy men. Serum acylated ghrelin was measured before and after a 7-day overfeeding challenge in 68 young men. Surprisingly, fasting ghrelin concentration was significantly increased in response to the positive energy challenge in the entire cohort. Based on our finding and the literature, we hypothesize that this increase may counteract the rising insulin resistance. At baseline, there were no significant differences in circulating acylated ghrelin concentration between normal weight, overweight, and obese men. However negative correlations were observed between ghrelin and BMI, in normal weight and overweight subjects. The baseline acylated ghrelin concentration correlated with change in weight and BMI in opposite directions in overweight and obese subjects. Thus future studies are warranted to understand the mechanistic pathway of ghrelin to further elucidate its role in energy homeostasis and human obesity.
